# Dexamethasone Inhibits Podocyte Apoptosis by Stabilizing the PI3K/Akt Signal Pathway

**DOI:** 10.1155/2013/326986

**Published:** 2013-04-24

**Authors:** Yu-Shengyou, Yu Li

**Affiliations:** Department of Pediatrics, Guangzhou First People's Hospital, Affiliate to Guangzhou Medical College, Guangzhou, Guangdong 510180, China

## Abstract

Corticosteroids like dexamethasone (DEX) are well-established treatments for the glomerular disease that sustain renal function, at least in part, by protecting podocytes from apoptotic death. In this study, we found that PAN causes abnormal expression of the PI3K-binding protein CD2AP, reducing PI3K/Akt signaling and promoting podocyte apoptosis. In contrast, DEX restores CD2AP-PI3K-Akt-GSK3**β** signaling, which promotes the activity of antiapoptotic proteins and inhibits the activity of proapoptotic proteins. In addition, we also found that CD2AP was aberrantly colocalized with p85. Normal CD2AP mRNA expression and subcellular protein distribution were maintained in the PAN + DEX group, and DEX coapplication also reduced CD2AP-p85 colocalization. PAN evoked a concentration-dependent decrease in p-Akt and p-GSK3**β** expressions, with p-Akt expression reaching a nadir at 15 min and p-GSK3**β** expression at 30 min. DEX treatment induced a concentration-dependent reversal of PAN-induced p-Akt and p-GSK3**β** downregulation. The PI3K inhibitor LY294002 blocked p-Akt and p-GSK3**β** expressions in podocytes. Cells treated with PAN exhibited a significantly higher apoptosis rate than untreated or vehicle-treated cells. Furthermore, LY294002 exacerbated PAN-induced apoptosis. DEX cotreatment caused a significant concentration-dependent decrease in PAN-induced apoptosis. These results strongly suggest that DEX protects podocytes by stabilizing the expression and subcellular distribution of CD2AP and by maintaining the expression of phosphor-activated Akt and GSK3**β**.

## 1. Introduction

Corticosteroids are widely used to treat glomerular disease. Recent studies demonstrated functional receptors for corticosteroids on glomerular podocytes [1], suggesting that clinical effects may be mediated by the rescue of podocyte function. Corticosteroids like DEX increase the stability of the cytoskeletal protein F-actin in podocytes and inhibit PAN-induced podocyte apoptosis. The cytoskeletal-associated protein CD2AP regulates cytoskeletal structure and cell morphology by directly binding to actin and a variety of membrane anchoring proteins. DEX improves podocyte survival by upregulating the expressions *α*-tubulin, CD2AP, and the transmembrane slit diaphragm protein nephrin. In addition, DEX downregulates the expression of vascular endothelial growth factor (VEGF) [2]. Wada et al. [3] found that DEX inhibited PAN-induced podocyte apoptosis by downregulating the expression of p53, upregulating Bcl-xL, and delaying the translocation of apoptosis-inducing factor (AIF) from cytoplasm to nucleus. Wada et al. [4] also found that PAN decreased the phosphorylation of extracellular signal-regulated kinase (ERK) and that DEX reversed this effect.

However, there have been no reports examining the direct effects of DEX on intracellular signaling pathways that may contribute to this antiapoptotic effect in podocytes. The PI3K/Akt signal pathway is widely distributed in eukaryotes and plays essential roles in growth, differentiation, proliferation, and survival. It is possible that DEX and other corticosteroids protect podocytes and improve proteinuria by activating the PI3K/Akt signaling pathway, but this has yet to be demonstrated. The purpose of this study was to determine if phosphoactivation of PI3K/Akt and the downstream target GSK3*β* is necessary for the anti-apoptotic effects of DEX against PAN-induced apoptosis in cultured mouse podocytes.

## 2. Materials and Methods

### 2.1. Cell Culture

Conditionally immortalized mouse podocyte clone (a kind gift from Professor Peter Mundel, USA) was cultured at 33°C in RPMI-1640 containing 10% fetal bovine serum (Gibco, Gaithersburg, MD, USA), 100 U/mL penicillin/streptomycin, and 10 U/mL of mouse recombinant r-interferon (PEPRO Tech, London, UK) and then shifted to 37°C for differentiation by removal of r-interferon which had typical character of mature podocyte after two weeks, In the studies described below, all experiments were performed in growth-restricted podocytes and repeated three times.

### 2.2. RT-PCR Analysis

Total RNA was extracted from podocyte using TRIzol Reagent according to the manufacturer's instruction, and the RNA concentration was determined after the sample was dissolved in diethylpyrocarbonate-treated water. Isolated RNA (1 *μ*g) of each sample was subjected to reverse transcription by using Rever Tra Ace (TOYOBO Co., Japan) according to the manufacture's protocol. The resulting cDNA (3 *μ*L) was used for PCR amplification. The sequence-specific primers were designed and synthesized by Shanghai Invitrogen Biotechnology Co, Ltd. Primers used were as follows: CD2AP upstream and downstream primers, respectively, were, Forward: 5-GCTCCTTCCTGACCCACT-3. Reverse: 5-TAAAAATACCCACCGCCA-3, product length being 238 bp; GAPDH upstream and downstream primers were,  Forward: 5-GGTGAAGGTCGGTGTGAACGGAT-3, Reverse: 5-CCACTTTGCCACTGCAAATGGCAG-3 product length being 118 bp. The PCR amplification was started with 2 min of denaturation at 95°C, which was followed by 34 cycles (for GAPDH, 30 cycles) of denaturation at 95°C for 30 s, annealing at 58°C for 30 s (GAPDH, 55°C for 30 s), and polymerization at 72°C for 45 s (GAPDH, 30 s). The final extension lasted 7 min at 72°C and then ended at 4°C. PCR products (5 *μ*L) were separated on 1% ethidium bromide-stained agarose gels and later scanned with gel imaging system (Bio Rad Company A). Independent experiment was repeated 3 times.

### 2.3. Western Blot Analysis

Podocytes were lyzed in the buffer containing 1% Tritonx-100, 150 mM NaCl, 1 mM EDTA, 50 mM Tris-HCl (pH 7.7), 1 mM NaF, 1 mM NaVO3, and a protease inhibitor cocktail (Sigma Chemical Co.). Seventy-five micrograms of total protein was loaded to run 8% sodium dodecyl sulfate-polyacrylamide gel electrophoresis (SDS-PAGE), and the gel was set up for transfer protein to nitrocellulose membranes (Sigma Chemical Co.). Then, the membranes were rinsed in a Tris-buffered saline with 0.02% Tween 20 (TTBS) and followed by immersing in 5% low-fat milk. Subsequently, the membranes were incubated with rabbit anti-first antibody (Sigma Chemical Co.), mouse anti-GAPDH antibody (Sigma Chemical Co.). After rinsing three times with TTBS, the membranes were incubated with HRP-conjugated goat anti-rabbit or mouse IgG (Sigma Chemical Co.) for 45 min at room temperature and then developed using ECL chemiluminescence reagent (Sigma Chemical Co.). The specific protein bands were scanned and quantitated using densitometry in relation to the GAPDH, western blotting detection system (GE Healthcare, Chalfont St. Giles, UK). We repeated each western blot analysis using protein from three different and separate experiments.

### 2.4. Immunofluorescence

First, antibody was fixed with 4% paraformaldehyde then permeabilized and blocked with 0.3% TritonX-100 and 5% bovine serum albumin. The primary antibody, rabbit anti-first antibody (Sigma Chemical Co.), was applied for overnight at 4°C. FITC-conjugated goat anti-rabbit or mouse IgG (Sigma Chemical Co.) and the nuclei dye Hoechst was used for 45 min at room temperature. Finally, the coverslips were mounted and images were taken by using an immunofluorescence microscope (Zeiss, Germany). we counted at least 200 nuclei in triplicate in each experiment.

### 2.5. Confocal Fluorescence Microscopy

Podocytes were cultured on glass slides and exposed to experimental conditions. Cells were fixed with 4% paraformaldehyde and permeabilized with 0.1% Triton-X for 5 minutes. Blocking was performed with 10% fetal bovine serum. Primary antibody was incubated at 4°C for 12 hours. Secondary antibody was incubated for 1 hours at room temperature. Cells were visualized by confocal microscopy. Blu-ray was detected with Zeiss LMS-inverted confocal microscope equipped with a 488 nm laser and with a 543 nm laser for green, using a Zeiss ×40 objective. Laser power and photomultiplier setting were kept identical for all samples to make the results comparable. Images were recorded and analyzed with the Zeiss LMS510 software (EMBL, Germany).

### 2.6. Statistical Analysis

Data were reported as mean ± SD with *n* equal to the number of experiments. Statistical evaluation was performed using a one-way ANOVA (two-sided test) followed by LSD (equal variances assumed) or Dunnett's T3 (equal variances not assumed) for post hoc test between two groups and also using the nonparametric tests (Mann-Whitney *U*-test) as a posttest. Values of *P* < 0.05 were considered as a statistical significance.

## 3. Results

### 3.1. Changes in the Expression and Distribution of CD2AP mRNA

**Figure 1:**
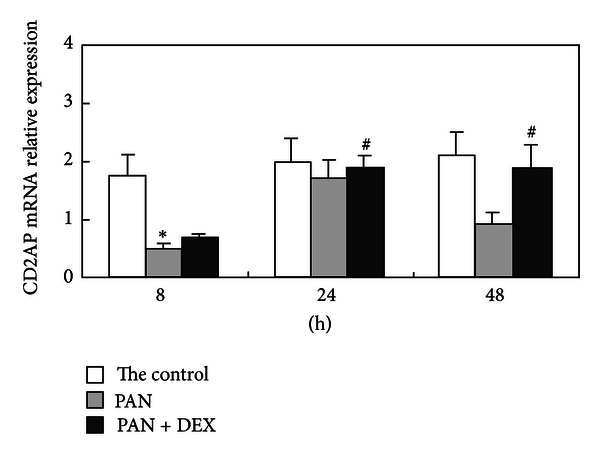
Changes in relative expression of CD2AP mRNA (CD2AP/GAPDH) for the control group, PAN-treated group, and PAN + DEX group at different time points. Note: By single factor analysis of variance, PAN-triggered group versus control group, *P < 0.05; DEX-treated group versus PAN group, ^#^ *P* < 0.05.

The expression of CD2AP mRNA in podocytes was significantly decreased 8 h after the application of PAN compared to expression of the internal reference GAPDH, and this downregulation was maintained for up to 48 h in PAN. Downregulation of CD2AP was reversed by DEX coapplication. Expression of CD2AP mRNA was significantly higher in cultures treated with PAN + DEX compared to cultures treated with PAN alone at 24 h and 48 h (*P* < 0.05); indeed, CD2AP mRNA expression in PAN + DEX cultures was not significantly lower than in vehicle-treated control cells (Figure 1).

### 3.2. Changes in Colocalization of CD2AP and p85

**Figure 2:**
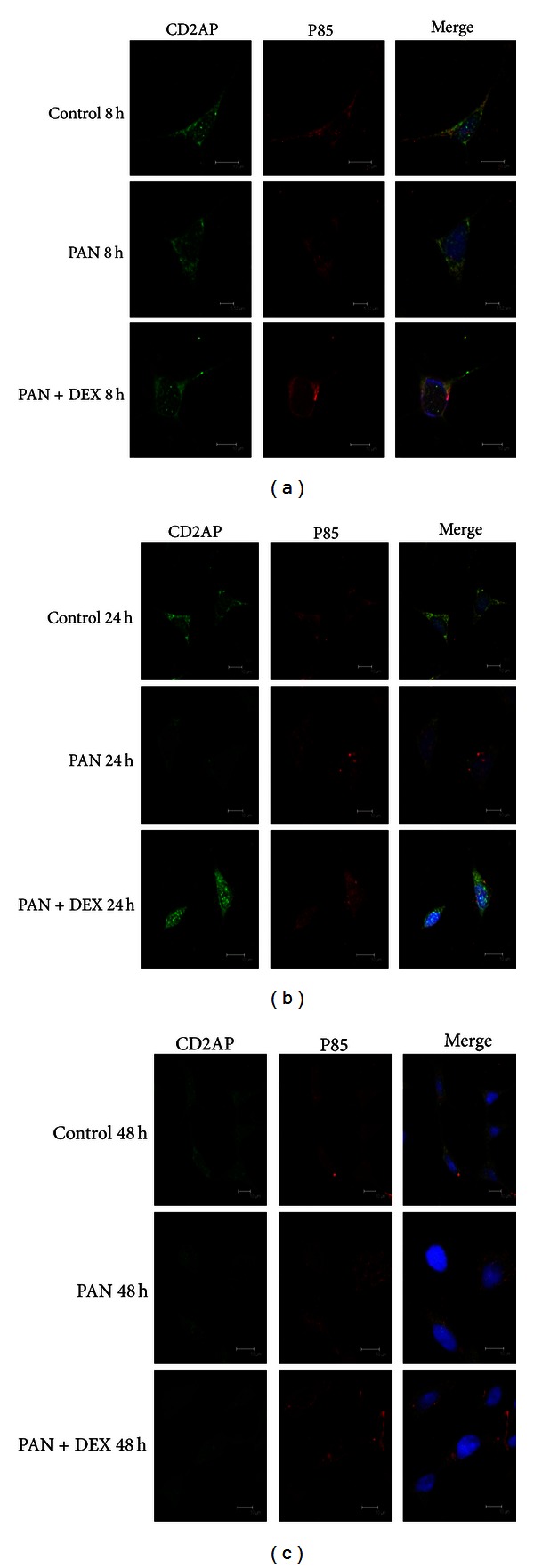
Changes in the co-localization of CD2AP and p85 in control cultures, PAN-treated cultures and PAN + DEX co-treated cultures as revealed by immunocytochemistry under confocal laser scanning microscopy (600x). In control cultures, significant overlap between the green fluorescent staining of CD2AP and the red fluorescent staining of p85 was observed in the cytoplasm, plasmamembrane, and nucleus. PAN-treated cultures showed decreased overlap in the cytoplasm at 24 and 48 h, but enhanced co-staining of the nuclear envelope and nucleus. Reduced nuclear fluorescence overlap at 24 h and 48 h in PAN + DEX co-treated cells.

In control, immunofluorescence examination revealed that CD2AP was evenly distributed in the nuclear envelope, cytoplasm, and plasmamembrane. Following PAN treatment, however, CD2AP was distributed in granules within the cytoplasm, perinuclear region, and nucleus, but was largely absent from the plasma membrane and most regions of the cytoplasm. DEX cotreatment reversed these changes in CD2AP distribution over time. In PAN + DEX cultures, CD2AP was distributed over a larger area of cytoplasm and plasma membrane, although granules were still observed in the cytoplasm 48 h after PAN + DEX treatment. In control cultures, overlap between CD2AP and the PI3K subunit p85 was observed in the nuclear envelope, cytoplasm, and plasma membrane. PAN-treated cultures exhibited significantly greater fluorescence overlap in the nucleus, and this change in subcellular distribution was reversed by DEX cotreatment; DEX treatment significantly enhanced fluorescence overlap in the perinuclear region and reduced overlap in the nucleus (Figure 2).

### 3.3. Decreasing Akt and GSK3*β* Phosphorylation by PAN and Reversal by DEX

**Figure 3:**
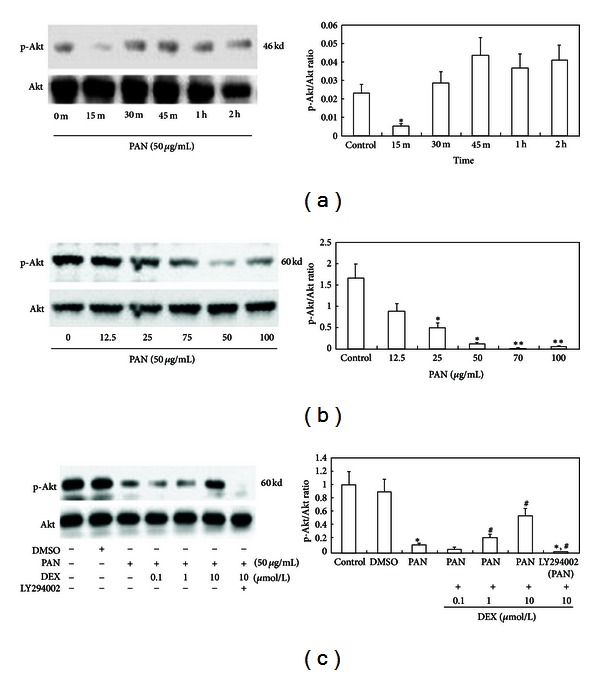
Changes in p-Akt/Akt induced by PAN, PAN + DEX, and LY294002 in podocytes. (a) p-Akt/Akt after 0, 15, 30, 45, 60, and 120 min of PAN (50 *μ*g/mL). (b) Concentration-dependence of PAN-induced changes in p-Akt/Akt as measured at 15 min after treatment with 0, 12.5, 25, 50, 70, or 100 *μ*g/mL PAN. (c) Reversal of the PAN response by DEX co-treatment and effect of LY294002 at 30 min. Lane 1: Control group, Lane 2: DMSO (0.02%), Lane 3: PAN (50 *μ*g/mL), Lanes 4–6: PAN (50 *μ*g/mL) + DEX (0.1, 1, or 10 *μ*mol/L), Lane 7: Pretreatment with LY294002 for 1 hour + PAN (50 *μ*g/mL) + DEX (10 *μ*mol/L) for 30 min. Lysates from podocyte treatment groups were collected for Western blot examination to detect the expression of p-Akt and total Akt. *P < 0.05 and **P < 0.01 compared to the control group; ^#^ *P* < 0.05 compared to PAN-treated group; in comparison with DEX-treated group *, ^#^ *P* < 0.01.

PAN induced both concentration- and time-dependent changes in the expression of p-Akt as revealed by Western blotting (Figure 3). In the presence of PAN (50 g/mL), podocyte p-Akt reached a nadir at 15 min. As little as 12.5 g/mL caused a significant decreased in p-Akt expression and expression decreased progressively as PAN concentrations increased from 12.5 to 50, 70, and 100 *μ*g/mL. Similar to the effect on CD2AP expression and subcellular distribution, the effect of PAN on Akt phosphorylation was reversed by DEX cotreatment and the reversal was DEX concentration dependent. The phosphorylation of Akt in PAN + DEX group podocytes approached that of control cultures. Pretreatment with LY294002 (25 M) for 1 h almost completely blocked Akt phosphorylation (Figure 3).

### 3.4. PAN-Induced Suppression of p-GSK3*β*

**Figure 4:**
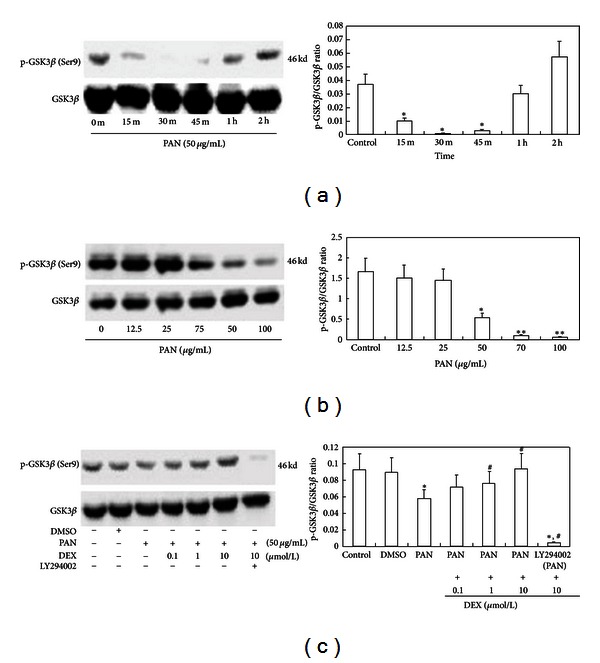
Suppression of GSK3*β* phosphorylation by PAN and reversal by DEX. (a) p-GSK3*β*/GSK3*β* at 0, 15, 30, 45, 60, and 120 min in PAN (50 *μ*g/mL) as revealed by Western blotting. (b) Concentration dependence of the PAN effect as indicated by Western blots after 30 min treatment with 0, 12.5, 25, 50, 70, and 100 *μ*g/mL PAN. (c) Reversal by DEX co-treatment and effect of LY294002. Lane 1: Control group, Lane 2: DMSO (0.02%), Lane 3: PAN (50 *μ*g/mL), Lanes 4–6: PAN (50 *μ*g/mL) + DEX (0.1, 1, and 10 *μ*mol/L), Lane 7: One hour pretreatment with LY294002 before PAN (50 *μ*g/mL) + DEX (10 *μ*mol/L) for 30 min. *P < 0.05, **P < 0.01 compare to control group; ^#^ *P* < 0.05 compared to the PAN-treated group; ^#^ *P* < 0.01 compared to the DEX-treated group.

PAN treatment also exerted concentration- and time-dependent changes in the expression of p-GSK3*β*, with the largest decrease in p-GSK3*β* expression observed at 30 min after PAN application. PAN decreases podocyte p-GSK3*β* at 12.5 *μ*g/mL, and expression progressively decreased as PAN concentrations increased (50, 70, and 100 *μ*g/mL). The effect of PAN was reversed by DEX (10 *μ*mol/L) co-treatment. Expression of p-GSK3*β* was almost completely blocked by LY294002 (25 mmol/L) for 1 h (Figure 4).

### 3.5. PAN-Induced Apoptosis and Rescue by DEX Cotreatment

**Figure 5:**
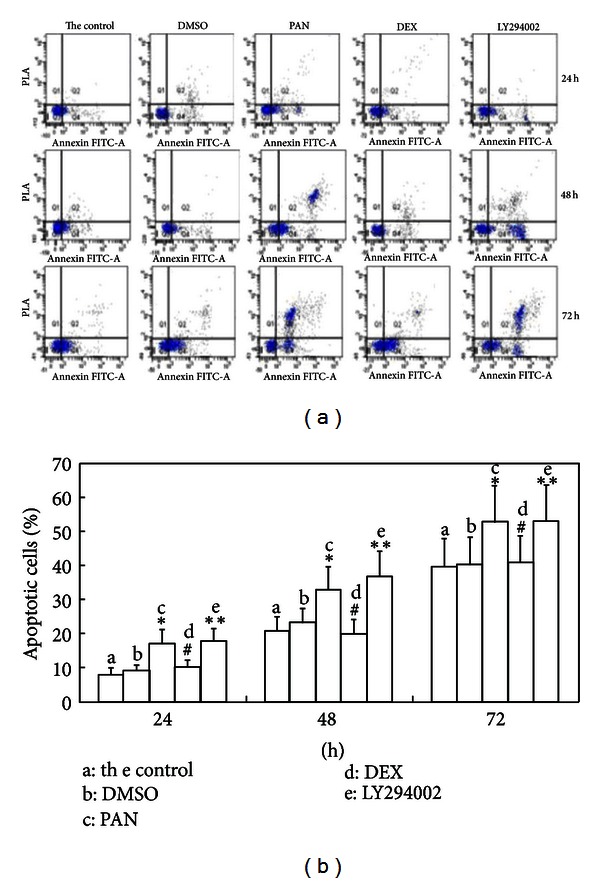
Apoptosis in the control group, DMSO group, PAN group, PAN + DEX group, and the LY294002-pretreated group at different time points (24, 48, and 72 h) as revealed by flow cytometry. Note: Compared to the control group, *P < 0.05; compared to PAN-treated group, ^#^ *P* < 0.01. The DMSO group was treated with 0.02% DMSO, the PAN-treated group with 50 *μ*g/mL, the PAN + DEX group with 50 *μ*g/mL PAN + 10 *μ*mol/L DEX, and the LY294002-pretreated group were pretreated with LY294002 for one hour and then treated with 50 *μ*g/mL PAN + 10 *μ*mol/L DEX.

PAN-treated podocytes showed greatly enhanced apoptosis after 24 h treatment compared to control and vehicle-treated (0.02% DMSO) cultures. Apoptosis was even higher after 48 h of PAN treatment and most podocytes were apoptotic by 72 h. The PAN + DEX group showed significantly lower apoptosis rates than the PAN-treated group at all time points. Pretreatment with LY294002 exacerbated apoptosis at (Figure 5).

## 4. Discussion

Podocytes are highly differentiated, morphologically complex epithelial cells consisting of the cell body, primary process, and a large secondary process or foot process. Podocytes are an important structural and functional component of the glomerular filtration membrane and the last protective barrier against protein leakage. Podocyte impairment is a key pathogenic even in the initiation and development of glomerular diseases associated with proteinuria. In the early stages of glomerular disease, there are significant changes in anatomic relationship between adjacent podocyte foot processes and between foot processes and the basement membrane, resulting in enhanced protein permeability resulting in proteinuria.

Podocyte apoptosis can be induced by PAN, angiotensin-II (Ang II), cyclosporine A, and by stretching. It is generally believed that podocytes are terminally differentiated nonregenerative cells with limited healing capacity. Thus, apoptosis leads to an irreversible decrease in the number of podocytes. The filtration barrier becomes incomplete, allowing blood proteins to penetrate this filtration membrane. The recent discovery of podocyte slit diaphragm-associated molecules like nephrin has led to a greater emphasis on the molecular mechanisms and signal pathways that regulate glomerular permeability. Recent studies on monogenetic inherited nephrotic syndrome (NS) and primary NS have revealed many protein molecules specifically expressed in podocytes, such as nephrin, CD2AP, podocin, and TRPC [5–7]. These podocyte-associated proteins are regulated by and activate signal pathways that maintain the normal structure and function of podocytes and the slit diaphragm.

The phosphoinositide-3-kinases (PI3Ks) specifically catalyze the phosphorylation of the hydroxyl group on the 3 position of phosphatidylinositol (PI). Normally, PI3Ks catalyze formation of the lipid products 3,4-diphosphate phosphatidylinositol (PI(3,4)P2) and 3,4,5-triphosphate phosphatidylinositol (PI(3,4,5)P3), both of which act as second messengers to activate the downstream signaling molecules protein kinase B (PKB/Akt) and glycogen synthesis kinase-3*β* (GSK-3*β*). Many downstream target proteins are then activated through Akt- and GSK-3*β*-mediated phosphorylation. These signaling cascades ultimately regulate such diverse cellular processes as proliferation, differentiation, survival, and translocation. The serine/threonine kinase Akt is central to PI3K-initiated cell signaling and is essential in such physiological and pathological processes such as angiogenesis, telomerase activation, and cell invasion, as well as in the modulation of cell cycle regulation and cell apoptosis.

The PI3K-Akt-GSK3*β*1 pathway is involved in the initiation and development of human renal diseases. Transforming growth factor *β*1 (TGF*β*1) acts on renal tubular epithelial cells to increase the phosphorylation of Akt, which results in reduced GSK3*β* kinase activity, promoting the transcription of proteins associated with the epithelial-mesenchymal transitional (EMT) [8]. The CD2AP protein contains one binding site for actin, one sequence abundant in proline, and three continuous SH3 (Src homology 3) domains. Interaction between the C terminal of CD2AP and the intracellular C terminal of nephrin stabilizes the slit diaphragm. In addition, the interaction of CD2AP with the cytoskeleton is regulated by receptors on the surface of podocytes [9]. Decreased expression of CD2AP was observed in a PAN-induced rat model of nephropathy [10, 11], while increased expression of CD2AP was observed in an adriamycin-induced nephrotic (ADN) rat model [12]. Despite reciprocal changes in expression, abnormal subcellular CD2AP distribution was observed in both models, suggesting that changes in the molecular interactions of CD2AP with other proteins rather than expression levels per se mediate kidney injury in response to PAN and adriamycin. 

Huber et al. [13] found that p85 immunoprecipitated with both CD2AP and nephrin in transfected HEK293T cells, indicating the presence of a nephrin-CD2AP-p85 complex. Akt, the downstream target molecule of PI3K, is activated in HEK293T cells overexpressing nephrin and CD2AP. When transfected with nephrin cDNA, conditionally immortalized podocytes showed increased Akt activity. Wortmannin, a specific inhibitor of PI3K, can completely block the activity of Akt. Therefore, it is possible that the interaction between CD2AP, nephrin, and p85 promotes the translocation of PI3K to the plasmalemma, activating the PI3K/Akt signaling pathway. Fan et al. [14] found that gene knockdown of CD2AP, nephrin, or podocin led to abnormal expressions of PI3K and Akt. Huber et al. [15] also found that podocin overexpression in HEK293 cells promoted nephrin phosphorylation, activation of p38MAPK, and increased transcription factor AP-1 activity. Woroniecki et al. [16] found that TGF-*β* expression was increased by 1.5-fold in CD2AP−/− animals. When stimulated by TGF-*β*, PI3K and ERK1/2 were rapidly phosphorylated to an early peak in wild-type cells, while CD2AP−/− podocytes showed delayed peak PI3K/ERK1/2 phosphorylation in response to TGF-*β*. Thus, it is suggested that CD2AP is involved in the TGF-*β*-induced activation of PI3K and ERK1/2. Tossidou et al. [17] activated growth factor receptor tyrosine kinases (RTKs) in podocytes and observed disordered PI3K/Akt and ERK signaling responses. Xavier et al. [18] found that CD2AP can directly interact with the TGF-*β* receptor subunit TbetaRI at the cytoplasmic tail and mediate the interaction between Tbeta RI and the PI3K regulatory subunit p85, activating the PI3K/Akt signal pathway to inhibit Tbeta-induced podocyte apoptosis. Zhu et al. [19] transfected podocytes with rat nephrin and podocin genes and observed the phosphorylation of the PI3K/Akt pathway as well as increased activity of Rac1. Huber et al. [20] demonstrated that lipid raft complex of podocin can recruit the ion channel protein TRPC6 to the SD and regulate its functions. Zhang et al. [21] suggested that Ang II-induced podocyte apoptosis might be related to TRPC6 upregulation, ERK activation, and NF-*κ*B translocation. Wada et al. [4] suggested that DEX exerts antiapoptotic effects by stabilizing ERK pathway.

Podocyte damage is also related to podocyte apoptosis caused by the inhibition of the Akt pathway [22]. Xiao et al. [23] observed significant podocyte apoptosis, a decreased p-Akt/Akt ratio, and increased activity of the TGF pathway in the PAN nephropathy rat model, suggesting that PAN induces podocyte apoptosis by activating TGF-*β*/Smad while blocking the PI3K/Akt pathway. The study by Wada et al. [3] showed that apoptosis of cultured podocytes in vitro was significantly increased by PAN treatment (30 g/mL) for 48 h and that apoptosis was significantly decreased by DEX. They further found that PAN induces podocyte apoptosis by upregulating p53 and Bax proteins and downregulating antiapoptotic Bcl-2. In contrast, DEX inhibits PAN-induced damage by downregulating p53 and Bax expression while upregulating antiapoptotic Bcl-2 expression. The most recent study by Wada et al. [4] demonstrated that PAN also decreased phosphorylation of ERK in podocytes and that reduced p-ERK was inhibited by DEX, suggesting that DEX exerts antiapoptotic effects on podocytes by stabilizing the phosphorylation of ERK.

In this study, we observed a significant decrease in CD2AP mRNA expression over 48 hours of PAN treatment. In 24 hours, the subcellular distribution of CD2AP was significantly altered in the plasma membrane and cytoplasm. By 48 hours, we observed CD2AP translocation into the nucleus. Colocalization of CD2AP with p85 was also altered by PAN, with decreased fluorescence overlap and accumulation in nuclei. Thus, we suggest that PAN inhibits the transcription of CD2AP mRNA and decreases the synthesis of CD2AP protein, which leads to inadequate CD2AP in the slit diagram, impairing diaphragm integrity and normal binding between CD2AP and p85. To test whether abnormal binding between CD2AP and p85 interferes with the activation of the downstream molecule Akt, we treated podocytes with PAN (50 g/mL) and measured the expression of p-Akt protein at 0, 15, 30, 45, 60, and 120 min and found that the expression of p-Akt decreased significantly compared to the control group at 15 min. We also treated podocytes with 6 PAN concentrations (0, 12.5, 25, 50, 75, and 100 g/mL) and measured the expression of p-Akt at 15 min, finding that PAN evoked a concentration-dependent decrease in p-Akt that was statistically significant for high PAN concentration (75 and 100 g/mL). Furthermore, these high PAN concentrations also induced apoptotic cell death in most podocytes, strongly suggesting that p-Akt mediates apoptotic resistance in podocytes. We also observed a similar trend in p-GSK3*β*, finding decreased p-GSK3*β* at 15, 30, and 45 min (with a nadir at 30 min) and a similar concentration-dependent decrease in p-GSK3*β* expression, which was statistically significant at higher PAN concentrations (75 and 100 g/mL).

The bcl-2-associated death promoter Bad can form heterologous dimers with Bax or Bak, replacing the Bax in heterologous dimers such as Bcl-2/Bax and Bcl-xl/Bax in a concentration-dependent manner. We speculate that PAN decreases the transcription of CD2AP, leading to lowered expression and abnormal subcellular distribution, which interferes with the interaction between CD2AP and p85, thereby reducing PI3K/Akt signal activation and downstream activation of antiapoptotic factors. DEX can increase the transcription of CD2AP and the expression of proteins and promote localization to the slit diaphragm, thus maintaining the integrity of the SD. DEX also facilitates the normal binding between CD2AP and p85, which activates PI3K, Akt, and GSK3*β* and exerts antiapoptotic effects on podocytes. DEX also demonstrated a concentration-dependent inhibition of PAN-induced podocyte apoptosis and reversed PAN-induced downregulation of p-Akt and p-GSK3*β*. Indeed, the expression of p-Akt and p-GSK3*β* was near that of untreated control cultures when 10 g/mL DEX was added to 50 g/mL PAN. When pretreated with LY294002 for 1 h, the phosphorylation of Akt and GSK3*β* in podocytes was almost completely blocked. Moreover, DEX cotreatment reduced Bad expression compared to PAN alone. We suggest that DEX stabilizes the transcription and normalizes the subcellular distribution of CD2AP. The binding of CD2AP to p85 activates the PI3K/Akt signal pathway, which then phospho-activates and phospho-inhibits various downstream target proteins. When phosphorylated, Bad affinity to various Bcl-2 protein family members is altered. Bad phosphorylation facilitates that activity of anti-apoptotic proteins, thereby promoting the survival and apoptotic resistance of podocytes.

In summary, PAN causes abnormal expression of the PI3K-binding protein CD2AP, reducing PI3K/Akt signaling and promoting podocyte apoptosis. In contrast, corticosteroids like DEX restore CD2AP-PI3K-Akt-GSK3*β* signaling, which promotes the activity of anti-apoptotic proteins and inhibits the activity of proapoptotic proteins. Further studies are required to unravel the additional molecular details of this cytoprotective pathway for the treatment of glomerular diseases.
